# Editorial: Proteins and protein-complexes underlying mitochondrial structure-function and metabolism: implications in diseases

**DOI:** 10.3389/fcell.2024.1386787

**Published:** 2024-03-04

**Authors:** Mohammad Golam Sabbir, Nawab John Dar, Shahnawaz Ali Bhat, Hamad H. Alanazi, Jeff Perry

**Affiliations:** ^1^ Department of Psychology and Neuroscience, College of Psychology, Nova Southeastern University, Fort Lauderdale, FL, United States; ^2^ Alzo Biosciences Inc., San Diego, CA, United States; ^3^ Salk Institute for Biological Studies, La Jolla, IL, United States; ^4^ Department of Zoology, Aligarh Muslim University, Aligarh, India; ^5^ Department of Clinical Laboratory Science, College of Applied Medical Sciences-Qurayyat, Jouf University, Sakakah, Saudi Arabia; ^6^ Hope National Medical Center Duarte, Santa Clara, CA, United States

**Keywords:** Alzheimer’s, mitochondria, cholinergic receptor muscarinic 1, huntingtin, huntingtin associated protein 1, respirasome, Sumoylation, Ubiquitination

The mitochondrial proteome plays a pivotal role in maintaining cellular health and function. The human MitoCarta3.0 dataset has curated 1136 proteins with mitochondrial localization primarily determined through mass spectrometry of mitochondria isolated from fourteen tissues and assessment of protein localization via large-scale green fluorescence protein tagging followed by microscopy (Rath et al., 2020). Many of these proteins are integral components of large complexes that control various aspects of mitochondrial functions. Some examples of these complexes, regulating crucial mitochondrial functions, include Oxidative phosphorylation (OXPHOS) complexes, mitochondrial permeability transition pore (mPTP), translocase complexes, pyruvate dehydrogenase complex, and mitochondrial ribosomes. While a significant number of mitochondrial proteins have undergone functional characterization, limited knowledge exists regarding their organization into complexes, stability, and dynamic assemblies under varying cellular physiological conditions. Recently, there has been a growing trend in the scientific community towards high-resolution profiling of these protein complexes or complexomes. This involves their resolution using Blue Native Polyacrylamide Gel Electrophoresis or density gradients, followed by mass spectrometric characterization and functional profiling (Wittig and Malacarne, 2021; Cabrera-Orefice et al., 2022). A comprehensive analysis of such complexomes profile is crucial for understanding the structural organization, molecular mechanisms, and regulatory processes governing mitochondrial functions implicated in the pathophysiology of different diseases. The articles in this Research Topic focus on some aspects of mitochondrial complexomes and their role in human diseases.

This Research Topic comprises four articles: two original research articles by Sabbir et al. one research article by Lee et al., and a review paper by Wang et al. Sabbir et al. research highlights mouse brain tissue-specific (cortex *versus* hippocampus) differential effect of the deletion of Cholinergic Receptor Muscarinic 1 (CHRM1) gene on mitochondrial structure, function, and assembly of respiratory protein complexes. These articles extend Sabbir et al. recent retrospective study published in the Journal of Alzheimer’s Disease, which involved the analysis of CHRM1 protein abundance in a large cohort of *postmortem* human brain tissues derived from Alzheimer’s disease (AD) patients and non-demented individuals (Sabbir et al., 2022).

The authors found a significant decrease in CHRM1 proteins (>50% decrease compared to age-matched non-demented individuals) in the temporal cortex of AD patients (N = 74) which correlated with early death (Sabbir et al., 2022). However, a similar loss in the corresponding hippocampus tissues exhibited no such correlation. It was also established that the reduction of CHRM1 was not linked to a decrease in downstream components of the G protein-coupled receptor (GPCR) signal transduction pathway, suggesting it may not be due to cholinergic synapse loss (Sabbir et al., 2022). These findings suggest that the use of choline esterase inhibitors (ChEIs) may not be an effective therapeutic strategy for AD patients with severely downregulated CHRM1 protein in their brains. This is reflected in the presence of ChEI non-responsive patients (approx. 50%) (Babiloni et al., 2006). CHRM1, a subtype of muscarinic acetyl choline (ACh) receptor (mAChR), has been associated with AD pathogenesis due to its predominant expression in the cerebral cortex and hippocampus, two brain regions affected during the early stage of the progression of AD. Therefore, the authors used Chrm1 deleted mouse line to analyze different molecular and structural parameters of mitochondria in the cortical and hippocampal neuropils to investigate potential tissue-specific differences. Interestingly, the authors found that Chrm1 loss in the cortex leads to severe structural abnormalities (loss of cristae) and reduction in mitochondrial function (respiration); in contrast, hippocampal loss was found beneficial to mitochondrial functioning (improved respiration). This contrasting finding was further corroborated by the observations that Chrm1 loss in cortex *versus* hippocampus differentially alters respiratory protein complexes assembly, correlating with the post-translational modifications of the key proteins involved in oxidative phosphorylation. Furthermore, a recent study by Sabbir published in the Journal of Alzheimer’s Disease (Sabbir, 2024) reported the colocalization and comigration of N-terminal green fluorescence protein-tagged Chrm1 with red fluorescence protein-labelled mitochondria in cultured adult primary rat dorsal root ganglion neurons, suggesting a potential localization of Chrm1 protein with mitochondria. The study observed the presence of abnormally swollen mitochondria with a loss of cristae in the Chrm1-deleted mouse peripheral sensory neurons, indicating a role for Chrm1 in the regulation of peripheral neurons’ mitochondrial structure (Sabbir, 2024). The reported structural, physiological, and molecular phenotypes under brain tissue-specific loss of Chrm1 protein represent a novel discovery that will shape the direction of Alzheimer’s research.

In their review article, Wang et al. offer a comprehensive overview of the roles of ubiquitylation and SUMOylation in the functioning of endoplasmic reticulum (ER) and mitochondrial proteins and protein complexes. The review delves into their generation, interactions, and regulatory influence on various aspects of cardiomyocyte physiology, particularly mitochondrial bioenergetics. Beyond ligand-activated conformational changes, post-translational modifications of proteins emerge as crucial regulators of protein complex association and dissociation (Betts and Sternberg, 1999; Marsh and Teichmann, 2015). Ubiquitination and SUMOylation substantially increase the molecular weight of proteins by over 8 kDa, presenting challenges for recognition using conventional techniques like western blotting. The significance of these modifications is discussed in detail along with a comprehensive summary of their signaling implications. These insights offer valuable perspectives on Ubiquitin/SUMO conjugation/deconjugation system in ER and mitochondrial functioning as well as its potential therapeutic implications in cardiovascular system.

Lee et al. study focuses on L-asparaginase-induced formation of mPTP-associated protein complex involving Huntingtin Associated Protein 1 (HAP1), huntingtin (HTT), and ER-associated Inositol 1,4,5-Trisphosphate Receptor (IP3R). This process results in the release of ER calcium ion (Ca^2+^) reserves, inducing the transfer of Ca^2+^ into mitochondria. Subsequently, this evokes an increase in reactive oxygen species (ROS), leading to the induction of apoptosis in acute lymphoblastic leukemia cells. These finding hold relevance to Sabbir et al.’s work because both HAP1/HTT (Tang et al., 2003) and muscarinic (Luo et al., 2001) signaling regulates neuronal Ca^2+^ homeostasis, are involved in neurodegenerative diseases (Sabbir et al., 2022), and one study showed epigenetic modification downregulates CHRM1-mediated Ca^2+^ signaling in Huntington’s disease (Lee et al., 2013). Furthermore, Ubiquitin and ubiquitin-binding proteins are major constituents of neurotoxic protein aggregates in neurodegenerative diseases. Dysregulated mitochondrial function supported by ubiquitin-mediated protein degradation pathways is linked to neurodegenerative diseases (Schmidt et al., 2021). In this context, the research articles published in this special issue highlight the central theme of the role of proteins and protein complexes in regulating mitochondrial function and the implications of their dysregulation in health and diseases.
